# Myxoma of the Pulmonary Valve: A Case Report and Review of the Literature

**DOI:** 10.7759/cureus.100790

**Published:** 2026-01-04

**Authors:** Rafael Martins, José Moreira, Ana Rita Moura, Mariana Vasconcelos, Jorge Casanova

**Affiliations:** 1 Cardiothoracic Surgery, Unidade Local de Saúde de São João, Porto, PRT; 2 Faculty of Medicine, University of Porto, Porto, PRT; 3 Cardiology, Unidade Local de Saúde de Matosinhos, Porto, PRT; 4 Cardiology, Unidade Local de Saúde de São João, Porto, PRT

**Keywords:** cardiac imaging, cardiac valvular tumor, pulmonary valve myxoma, right ventricular outflow tract obstruction, surgical management

## Abstract

Pulmonary valve myxomas are exceptionally rare cardiac tumors, with only a few reported cases in the literature, and their nonspecific presentation and uncommon location create diagnostic and surgical challenges. We report the case of a 68-year-old woman who presented with mild exertional dyspnea; transthoracic echocardiography demonstrated a 12 × 17 mm mobile mass prolapsing through the pulmonary valve, and cardiac magnetic resonance confirmed a pedunculated tumor attached to the ventricular surface of the left pulmonary cusp, without evidence of distal embolization. The patient underwent surgical excision with en bloc resection of the involved cusp and pericardial reconstruction, followed by an uneventful postoperative recovery, and histopathological analysis confirmed the diagnosis of myxoma. Pulmonary valve myxomas may manifest with obstructive, embolic, or constitutional symptoms, with dyspnea from right ventricular outflow tract obstruction representing the most frequent clinical presentation; diagnosis relies heavily on multimodality imaging, and complete surgical excision remains the definitive treatment. This case highlights the extreme rarity of pulmonary valve myxomas and underscores the value of comprehensive imaging assessment and meticulous surgical technique to achieve full tumor resection and favorable clinical outcomes.

## Introduction

Cardiac myxomas are the most common primary cardiac tumors [1,2]. They are predominantly located in the left atrium (approximately 75%) and right atrium (15-20%), while ventricular involvement is uncommon, accounting for about 3-4% of cases in each ventricle [1,2]. Most myxomas originate from mesenchymal cells of the cardiac chambers and septa; however, in rare instances, they may arise from the cardiac valves and are therefore classified as valvular myxomas [2]. Among these, the mitral valve is most frequently affected, followed by the tricuspid and aortic valves, whereas involvement of the pulmonary valve is exceptional [2]. Pulmonary valve myxomas are exceedingly rare, with approximately 10 cases reported in the literature to date, and represent a distinct clinical entity associated with diagnostic and therapeutic challenges due to their unusual location and nonspecific presentation [1,3-8].

## Case presentation

**Figure 1:**
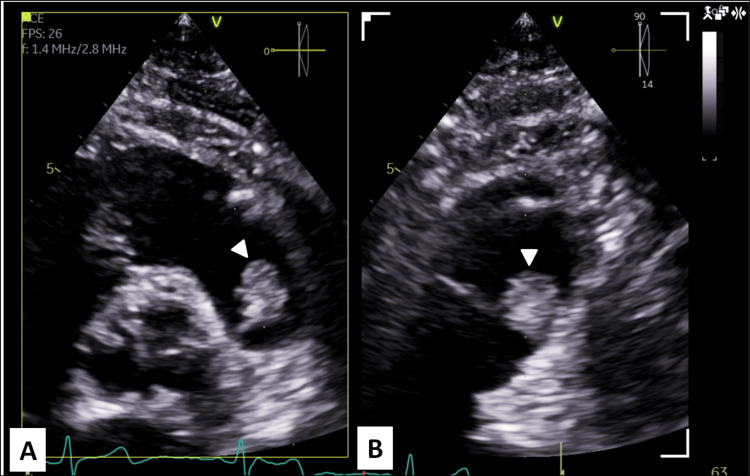
Parasternal Short-Axis Transthoracic Echocardiography (A) and (B) demonstre transthoracic echocardiographic parasternal short-axis views with multiplanar imaging, revealing a mobile, heterogeneous mass attached to the pulmonary valve (arrowheads).

**Figure 2:**
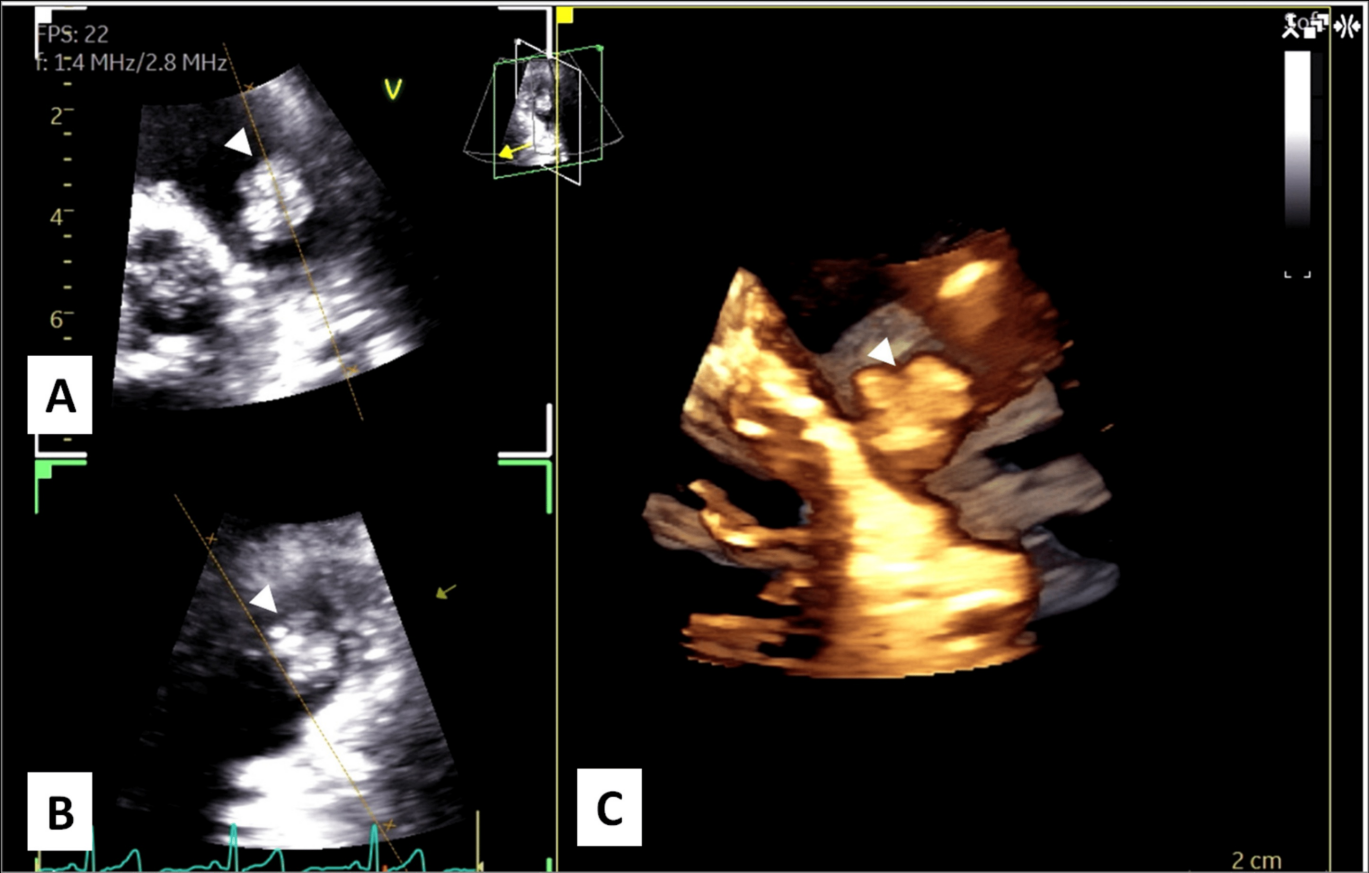
Transthoracic Echocardiography With Two-Dimensional and Three-Dimensional Imaging (A) and (B) demonstrate two-dimensional transthoracic echocardiographic views showing a mobile, heterogeneous mass (arrowheads) arising from the pulmonary valve and protruding into the right ventricular outflow tract during the cardiac cycle, while (C) demonstrates three-dimensional transthoracic echocardiographic reconstruction providing detailed spatial visualization of the lobulated mass attached (arrowheads) to the pulmonary valve, allowing improved assessment of its morphology and valve involvement.

A 68-year-old female patient was referred to our cardiac surgery outpatient clinic with a presumptive diagnosis of pulmonary valve myxoma, a rare form of valvular cardiac tumor [1,3]. At the referring hospital, she reported a three-month history of New York Heart Association (NYHA) class I heart failure [9]. Transthoracic echocardiography revealed a 12 × 17 mm irregular, friable mass with systolic to-and-fro motion across the pulmonary valve (Figures 1, 2). The remaining valvular structures appeared normal, and biventricular systolic function was preserved.

**Figure 3:**
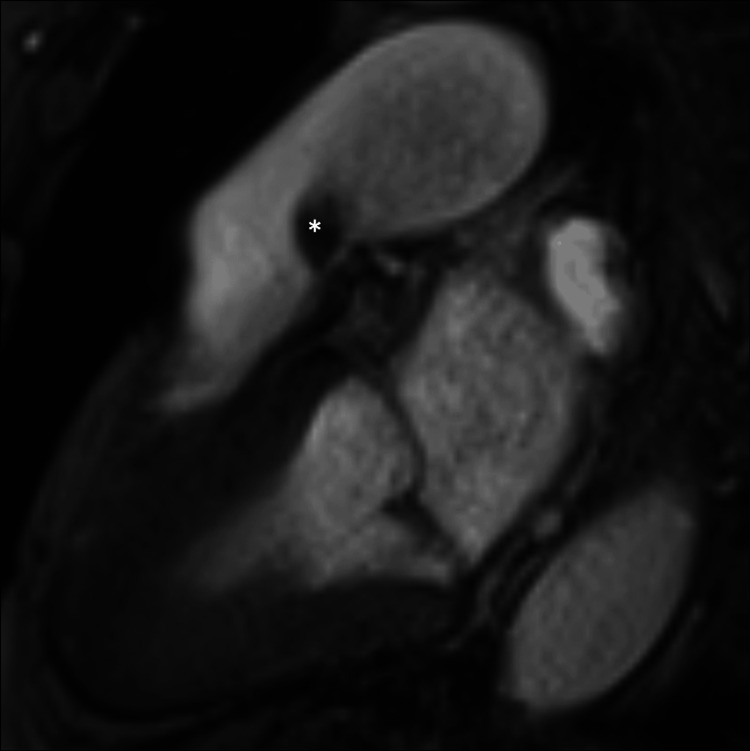
Cardiac Magnetic Resonance Cine SSFP Imaging Preoperative cardiac magnetic resonance cine steady-state free precession (SSFP) sequence acquired in systole demonstrating a pulmonary valve mass (*).

**Figure 4:**
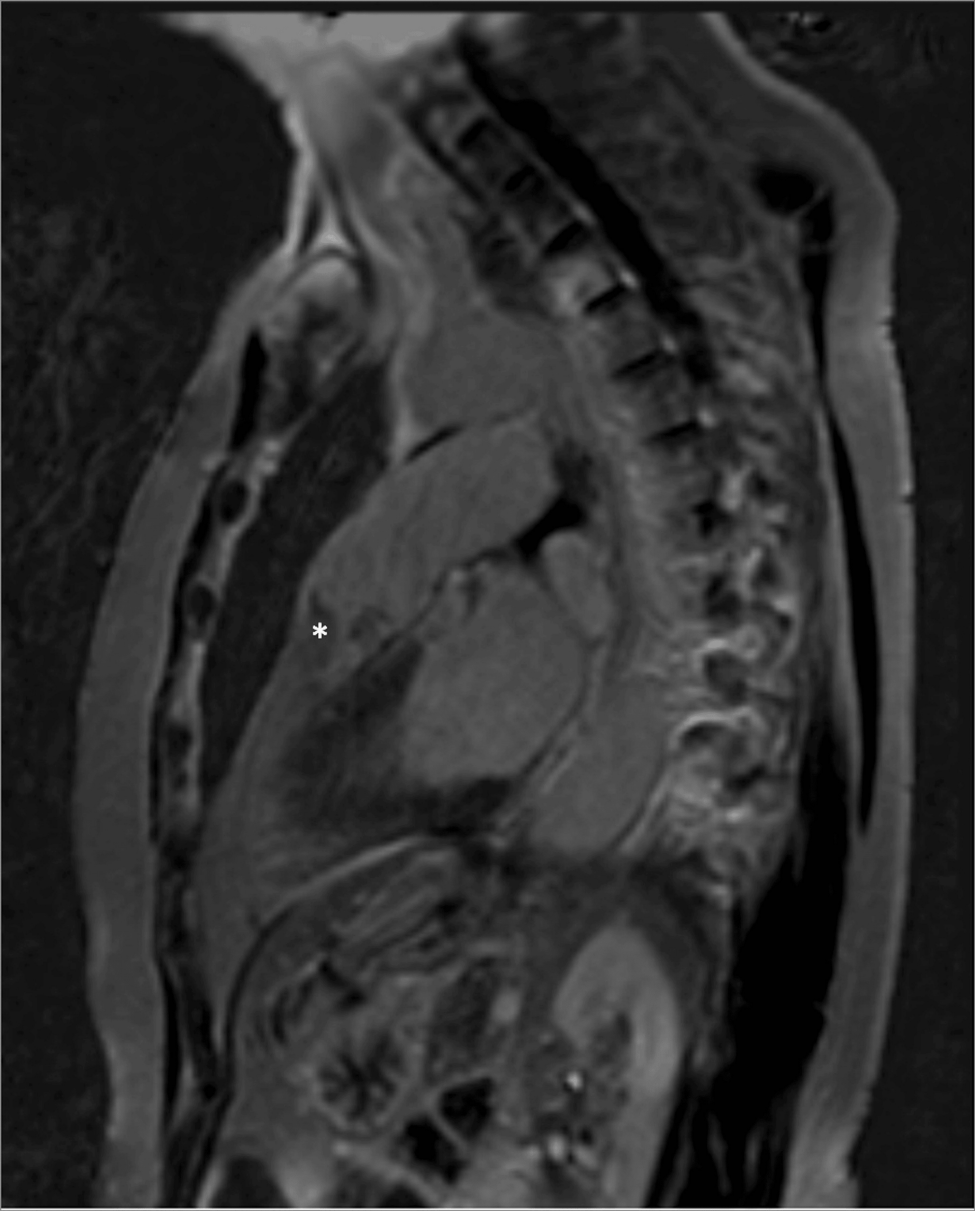
Cardiac Magnetic Resonance Late Gadolinium Enhancement Preoperative cardiac magnetic resonance imaging with late gadolinium enhancement demonstrating a pulmonary valve mass without significant contrast uptake (*).

**Figure 5:**
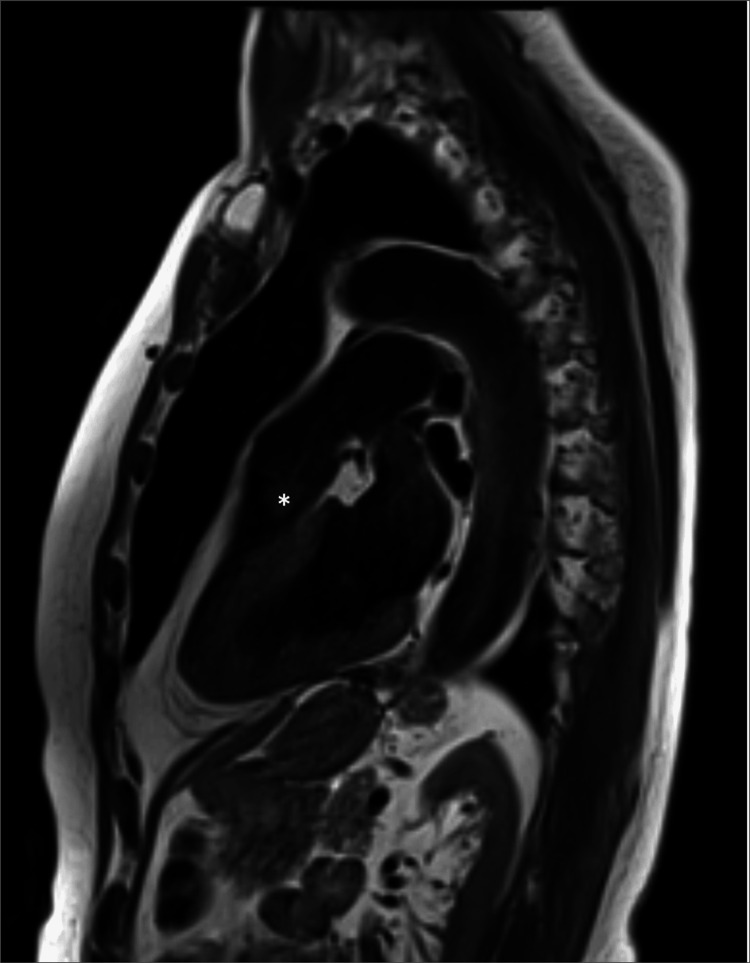
Cardiac Magnetic Resonance T1-Weighted Imaging Preoperative cardiac magnetic resonance T1 turbo spin-echo sequence demonstrating a pulmonary valve mass (*).

**Figure 6:**
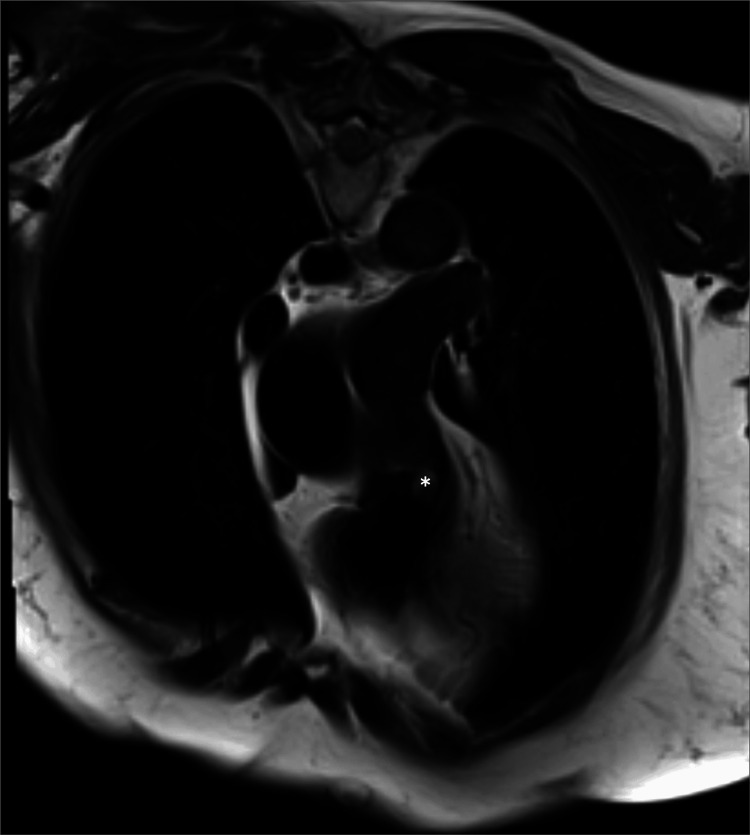
Cardiac Magnetic Resonance T2-Weighted Imaging Preoperative cardiac magnetic resonance T2 turbo spin-echo sequence demonstrating a pulmonary valve mass (*).

A cardiac magnetic resonance imaging revealed a pedunculated 15 mm mass originating from the ventricular surface of the left pulmonary valve leaflet, crossing the pulmonary valve in systole, suggestive of a myxoma or papillary fibroelastoma (Figures 3-6). A chest computed tomography scan excluded the presence of distal embolization. A positron emission tomography with fluorine-18 fluorodeoxyglucose (¹⁸F-FDG PET) scan did not reveal increased glycolytic metabolism. Consequently, the patient was referred for surgery.

At surgery, after median sternotomy, cardiopulmonary bypass was established through aorto-bicaval cannulation, and the patient was cooled to 32°C. After cardioplegic arrest, the pulmonary valve was exposed through an arteriotomy extending to the pulmonary annulus. A mass, as described, approximately 12 × 17 mm in size, with a 5 × 5 mm stalk attached to the ventricular surface of the left pulmonary leaflet, was found. En bloc resection of the cusp was performed, and a glutaraldehyde-fixed autologous pericardial patch was used to restore the pulmonary valve leaflet, followed by a bovine pericardial patch repair of the pulmonary trunk. After unclamping, sinus rhythm was restored, and cardiopulmonary bypass was easily discontinued. Transesophageal echocardiography confirmed normal pulmonary valve function and preserved biventricular function. The patient experienced an uneventful recovery and was discharged on the sixth postoperative day. At follow-up, she remained asymptomatic.

## Discussion

Pulmonary valve myxomas predominantly affect middle-aged adults, show a slight male predominance (male-to-female ratio of approximately 5:4), and demonstrate a bimodal age distribution that includes both pediatric and elderly patients [1].

Clinical manifestations of cardiac myxomas are traditionally categorized as obstructive, embolic, or constitutional, depending on tumor size, location, and friability [2]. In pulmonary valve myxomas, obstructive symptoms may result from the disproportion between tumor size and the relatively small effective orifice area of the pulmonary valve. Embolic manifestations can mimic thromboembolic pulmonary embolism, while nonspecific constitutional symptoms may occur, similar to myxomas arising in other cardiac chambers [1,2]. Compared with other locations, pulmonary valve myxomas are less frequently asymptomatic. Review of published cases indicates that dyspnea secondary to right ventricular outflow tract obstruction is the most common presentation, whereas other manifestations include cardiac murmur or incidental detection on echocardiography [1,3-8]. Less frequent presentations include syncope, right-sided heart failure, and complete atrioventricular block, while sudden death and asymptomatic detection remain exceptional findings [1].

Transthoracic echocardiography is the primary diagnostic modality for cardiac myxomas. However, cardiac magnetic resonance imaging or computed tomography is often required to differentiate myxomas from thrombi or vegetations and to assess tissue characteristics, tumor extent, and the presence of distal pulmonary embolization [1,4].

Surgical excision remains the treatment of choice and should aim for complete tumor removal with adequate margins to minimize the risk of recurrence [1,2]. In pulmonary valve myxomas, particular care is required to prevent intraoperative fragmentation and distal embolization. Surgical management frequently necessitates partial or complete resection of the pulmonary valve cusps, with subsequent valve repair or replacement and reconstruction of adjacent structures [1]. In the present case, resection of one pulmonary cusp followed by reconstruction with autologous pericardium and pulmonary trunk patch repair was required.

Postoperative echocardiographic follow-up is essential to assess pulmonary valve function and detect recurrence, as myxomas may recur even after complete excision. In pulmonary valve myxomas, recurrence has been reported in one of the few cases with available follow-up, likely related to incomplete primary excision [1]. Although postoperative mortality has been reported in a minority of cases, this appears to be more closely related to patient comorbidities and clinical status rather than tumor location itself [1,4,5]. Overall, prognosis after complete surgical removal is favorable in the majority of reported cases [6-8].

## Conclusions

Pulmonary valve myxomas are exceptionally rare cardiac tumors and may pose diagnostic challenges due to their unusual location and nonspecific clinical presentation. Multimodality imaging, particularly transthoracic echocardiography complemented by cardiac magnetic resonance imaging, is essential for accurate diagnosis and surgical planning. Complete surgical excision remains the treatment of choice and is associated with favorable outcomes when meticulous resection and appropriate valve reconstruction are achieved.
